# Structural Validity of the World Assumption Scale

**DOI:** 10.1002/jts.22348

**Published:** 2018-12-16

**Authors:** Vincent van Bruggen, Peter M. ten Klooster, Niels van der Aa, Annemarie J.M. Smith, Gerben J. Westerhof, Gerrit Glas

**Affiliations:** ^1^ Department of Psychology Health and Technology University of Twente Enschede the Netherlands; ^2^ Foundation Centrum ’45 | Partner in Arq Psychotrauma Expert Group Oegstgeest and Diemen the Netherlands; ^3^ Department of Philosophy Vrije Universiteit Amsterdam the Netherlands

## Abstract

The World Assumption Scale (WAS) is a frequently used measure in trauma research. The 32 items of the WAS are intended to represent eight assumptions about the benevolence of the world, the meaningfulness of events, and the worthiness of the self. Debate about the validity of the WAS is ongoing, particularly in terms of its empirical factor structure; some studies have confirmed a model of eight correlated factors whereas several other studies have not. The WAS items were administered to a clinical sample of patients who sought professional help because of posttraumatic complaints (*n* = 1,791) as well as a sample of healthcare professionals (*n* = 236). We split the clinical sample into three subsamples, then performed exploratory factor analysis using data from one subsample and tested the factor structure with confirmatory factor analysis using the other two subsamples. A consistent model of eight correlated factors was demonstrated, with almost all factors showing acceptable reliability, Cronbach's αs = .68–.84. We tested this factor model against data from the sample of healthcare professionals with increasingly stringent levels of invariance and found it to be scalar invariant (same structure, loadings, and thresholds). In a regression analysis, five factors showed significant associations with posttraumatic stress disorder (PTSD) symptoms, and two factors had unique associations with PTSD symptoms after we controlled for traumatic events: Self‐Worth, β = −.31; and Luck, β = −.15. Future research should aim to distinguish between different assumptions and their individual influences on posttraumatic complaints.

## Resumen

Spanish Abstracts by Asociación Chilena de Estrés Traumático (ACET)

Validez estructural de la Escala de Supuestos acerca del Mundo (World Assumption Scale).

La Escala de Supuestos acerca del Mundo (WAS en sus siglas en inglés) es una medida utilizada con frecuencia en la investigación del trauma. Los 32 ítems del WAS intentan representar ocho suposiciones sobre la benevolencia del mundo, el significado de los eventos y la dignidad del yo. El debate sobre la validez de la WAS está en curso, particularmente en términos de su estructura factorial empírica; algunos estudios han confirmado un modelo de ocho factores correlacionados, mientras que otros estudios no lo han hecho. Los ítems de WAS se administraron a una muestra clínica de pacientes que buscaron ayuda profesional debido a quejas postraumáticas (*n* = 1,791), así como una muestra de profesionales de la salud (*n* = 236).

Dividimos la muestra clínica en tres submuestras, luego realizamos un análisis factorial exploratorio utilizando datos de una submuestra y probamos la estructura factorial con un análisis factorial confirmatorio utilizando las otras dos submuestras. Se demostró un modelo consistente de ocho factores correlacionados, con casi todos los factores que muestran una confiabilidad aceptable, αs de Cronbach = .68 – .84. Probamos este modelo factorial con datos de la muestra de profesionales de la salud con niveles de invariancia cada vez más estrictos y se encontró que cumplía con la invarianza escalar (misma estructura, cargas y umbrales). En un análisis de regresión, cinco factores mostraron asociaciones significativas con los síntomas del trastorno de estrés postraumático (TEPT), y dos factores tuvieron asociaciones únicas con los síntomas de TEPT después de que controlamos los eventos traumáticos: autoestima, β = ‐.31; y suerte, β = ‐.15. Las investigaciones futuras deben apuntar a distinguir entre diferentes supuestos y sus influencias individuales en las quejas postraumáticas.

## 抽象

Traditional and Simplified Chinese Abstracts by the Asian Society for Traumatic Stress Studies (AsianSTSS)

簡體及繁體中文撮要由亞洲創傷心理研究學會翻譯

Traditional Chinese

標題: 世界假定量表的結構性效度

撮要: 世界假定量表(WAS)是創傷研究常用的測量工具。WAS裡的32個項目乃基於8項有關世界的善心、事件意義和自我價值的假設。然而, WAS的效度, 特別是它的實證因素結構, 一直備受爭議。有些研究驗證了一個有8種相關因素的模型, 但其他研究卻無法驗證。本研究對臨床樣本採用WAS。樣本包含因創傷後的問題尋求治療的人士(n = 1,791), 以及醫療專業人員(n = 236)。我們把臨床樣本分為3組子樣本, 對1組的數據進行探索性因素分析, 並以其餘兩組進行驗證性因素分析, 測試因素結構。結果與過往得出的8種相關因素模型一致, 而且近乎所有因素都有可接受的信度(Cronbach's αs = .68–.84)。我們再以醫療專業人員組成的樣本試驗這個因素模型。這次試驗具更高水平的不變性, 並發現模型具尺量不變性 (同一結構、負苛、閾限) 。迴歸分析顯示, 5種因素跟創傷後壓力症(PTSD)症狀有顯著關連;我們對創傷事件 (自我價值, β = ‐.31; 和運氣, β = ‐.15) 進行對照試驗後, 發現2種因素與PTSD症狀有獨特關連。未來研究應針對分辨各種假設分別對創傷後問題造成的影響。

标题: 世界假定量表的结构性效度

撮要: 世界假定量表(WAS)是创伤研究常用的测量工具。WAS里的32个项目乃基于8项有关世界的善心、事件意义和自我价值的假设。然而, WAS的效度, 特别是它的实证因素结构, 一直备受争议。有些研究验证了一个有8种相关因素的模型, 但其他研究却无法验证。本研究对临床样本采用WAS。样本包含因创伤后的问题寻求治疗的人士(n = 1,791), 以及医疗专业人员(n = 236)。我们把临床样本分为3组子样本, 对1组的数据进行探索性因素分析, 并以其余两组进行验证性因素分析, 测试因素结构。结果与过往得出的8种相关因素模型一致, 而且近乎所有因素都有可接受的信度(Cronbach's αs = .68–.84)。我们再以医疗专业人员组成的样本试验这个因素模型。这次试验具更高水平的不变性, 并发现模型具尺量不变性 (同一结构、负苛、阈限) 。回归分析显示, 5种因素跟创伤后压力症(PTSD)症状有显著关连;我们对创伤事件 (自我价值, β = ‐.31; 和运气, β = ‐.15) 进行对照试验后, 发现2种因素与PTSD症状有独特关连。未来研究应针对分辨各种假设分别对创伤后问题造成的影响。

Psychological sequalae of exposure to traumatic events has been an important point of interest in psychological research since at least the end of the 19th century (Weisæth, 2002). Human beings live in a threatening world in which a wide array of adverse events may happen, such as the loss of a loved one, personal injury, being detained, or becoming the victim of a man‐made or natural disaster. In general, people have the resilience to survive even the most adverse events, and most survivors are able to adapt to the changed circumstances and integrate the extreme experience into their cognitive framework (Herman, 1993). However, this process of adaption is not always successful, and a substantial percentage of trauma survivors develop mental health complaints, including posttraumatic stress disorder (PTSD) and/or depression (Kilpatrick et al., 2013; Stander, Thomsen, & Highfill‐McRoy, 2014).

Cognitions are one of the important factors in how individuals cope with traumatic events (Ehlers & Clark, 2000). Long‐, and often implicitly, held beliefs about the world and oneself prove invalid in the light of sudden misfortune. Janoff‐Bulman (1992) elaborated on this aspect of coping with trauma and developed the theory of shattered assumptions. The basic idea behind this theory is that individuals need a set of stable cognitions about the world that are helpful in predicting events and guide perception and decision making (Janoff‐Bulman, 1992). This might apply even more for abstract assumptions that are at the base of the meaning‐making system of an individual: “It is change in these most fundamental schemas, deeply embedded within our conceptual system, that is at stake in the case of traumatic life events” (Janoff‐Bulman, 1989, p. 116). Janoff‐Bulman distinguished eight basic world assumptions, such as Benevolence of the People, which is the assumption that other people are basically good, kind, helpful, and caring, and trustworthy; and Self‐Worth, which is an individual's assumption the he or she is a good, moral, worthy, and decent individual. These assumptions are consistently typified as “our assumptions,” and it is important to note that the theory of shattered assumptions is supposed to apply to people in general and not only to clinical populations. This is also apparent in the description of the development of these assumptions as part of a normal life course. According to Janoff‐Bulman (1992), people start life with a positive set of world assumptions, a result of the warmth and nurturing children normally receives in their first years. These assumptions are usually not changed by daily hassles and minor negative events, and when changes do occur, it is a slow and gradual process. However, in the case of a traumatic event, a person is confronted with the complex task of integrating shocking information that strongly contradicts the existing framework of positive assumptions. For example, the assumption that other people are basically trustworthy becomes problematic when one is personally confronted with aggressive acts by other people. When the task of integrating this traumatic information into existing assumptions is not resolved, the tension between an individual's world assumptions and the newly acquired information about the traumatic events continues, which might also give rise to psychopathology. It seems important that therapists are aware of the possibility of change in basic assumptions so they can pay proper attention to this aspect of posttraumatic suffering when necessary.

The possible effect of an assumption depends on its interrelatedness with other assumptions. For example, when someone has a strong belief that outcomes are distributed in accordance with personal efforts (i.e., the assumption of Controllability, per Janoff‐Bulman [1992]), this belief is only helpful together with the trust in one's own capability to make the necessary efforts (i.e., the assumption of Self‐Controllability). The eight assumptions can be categorized in three primary categories. The first category concerns the benevolence of the world, the second concerns the meaningfulness of events and how these are distributed, and the third concerns a person's own worthiness. Janoff‐Bulman (1989) also developed an accompanying measurement instrument, the World Assumption Scale (WAS), which includes 32 items intended to measure the eight world assumptions. The eight assumptions, three primary categories, and items included in the WAS can be found in Table 1.

**Table 1:** Primary Categories, Domains, and Items of the World Assumption Scale^a^

Primary Categories and World Assumptions	Item Numbers and Wording^b^
*Benevolence of the World*	
Benevolence of the Impersonal World (BW)	5: The good things that happen in this world far outnumber the bad. 9: There is more good than evil in the world. 30: If you look closely enough, you will see that the world is full of goodness. 25: The world is a good place.
Benevolence of the People (BP)	12R: People don't really care what happens to the next person. 2R: People are naturally unfriendly and unkind. 26: People are basically kind and helpful. 4: Human nature is basically good.
*Meaningfulness*	
Justice (J)	14: People will experience good fortune if they themselves are good. 19: By and large, good people get what they deserve in this world. 7: Generally, people deserve what they get in this world. 1: Misfortune is least likely to strike worthy, decent people.
Controllability (C)	11: People's misfortunes result from mistakes they have made. 20: Through our actions we can prevent bad things from happening to us. 22: If people took preventive actions, most misfortune could be avoided. 29: When bad things happen, it is typically because people have not taken necessary actions to protect themselves.
Randomness^c^ (R)	3R: Bad events are distributed to people at random. 6R: The course of our lives is largely determined by chance. 15R: Life is too full of uncertainties that are determined by chance. 24R: In general, life is mostly a gamble.
*Worthiness of the Self*	
Self‐Worth (SW)	8R: I often think that I am no good at all. 18R: I have a low opinion of myself. 31R: I have reason to be ashamed of my personal character. 28: I am very satisfied with the kind of person I am.
Self‐controllability (SC)	13: I usually behave in ways that are likely to maximize good results for me. 17: I almost always make an effort to prevent bad things from happening to me. 23: I take the actions necessary to protect myself against misfortune. 27: I usually behave so as to bring about the greatest good for me.
Luck (L)	10: I am basically a lucky person. 16: When I think about it, I consider myself very lucky. 21: Looking at my life, I realize that chance events have worked out well for me. 32: I am luckier than most people.

*Note*. ^a^Other labels can be found in literature; for example, World Assumptions Scale, or Assumptive World(s) Scale.

^b^ “R” next to an item number indicates that the wording of an item is opposite to the meaning of the domain it belongs to. The answers of these items have to be rescored.

^c^ Was also labeled “Chance” in the original paper by Janoff‐Bulman (1989).

In the past two decades, the WAS has become a central instrument in trauma research. We searched PsycINFO, PubMed, and Web of Science, using different and known wordings of “World Assumptions Scale”; this resulted in 164 unique publications about studies in which the WAS has been used. Most research involving trauma victims reported evidence for changes in assumptions about benevolence of the world and self‐worth (Kaler, 2010). Despite its popularity, several researchers have questioned the construct validity of the WAS (e.g., Coyle, 1995; Jeavons & Godber, 2005; Kaler et al., 2008). Evidence for the theory of shattered assumptions is mixed, and the contradictory findings might be caused, in part, by unanswered questions about the structural validity of the WAS. Researchers make different decisions with regard to scoring the WAS, with some using total scores and others scoring either the three primary categories, the eight subscales, a selection of these scales, or newly composed scales; this complicates comparisons of results and the drawing of overall conclusions. Several researchers have analyzed the structural validity of the WAS. The original structure in eight factors (or seven, when both benevolence factors were taken together [Janoff‐Bulman, 1992]) was able to be replicated in a confirmatory factor analysis (CFA) that used a sample of whiplash patients (Elklit, Shevlin, Solomon, & Dekel, 2007) but not by Kaler et al. (2008) in a sample of students. Several studies that have used exploratory factor analysis (EFA) have also been conducted. These studies have provided evidence for a five‐factor solution (Harris & Valentiner, 2002), a three‐factor solution (Jeavons & Greenwood, 2007), and various four‐factor solutions (Lilly, 2011; Littleton & Breitkopf, 2006; Rini et al., 2004). Until now, consensus about the factor structure has not been reached, which is problematic as a clear conclusion about the factor structure of the WAS is a prerequisite for drawing any conclusions about other aspects of its validity and future examination of the theory of shattered assumptions.

In this study, we aimed to contribute to the literature by thoroughly examining the structural validity of the WAS using both a large clinical sample and a sample with a different background. Because previously conducted studies showed different factor solutions, we started with an EFA in our clinical sample and then tested this solution using CFA in two different subsamples of this larger group. We expected that the results of the EFA would show different factors that were strongly related, as is often the case for measurement instruments of psychological constructs. For this reason, we used a bifactor model in our CFA to study the ratio between the specific factors found in EFA and a general underlying factor. We did not find previous studies that used a bifactor model to analyze the WAS, but bifactor modeling has proven useful before in studying related constructs, such as PTSD symptoms (e.g., Byllesby et al., 2017). Finally, we examined measurement invariance in a sample of healthcare professionals. We studied the relevance of distinguishing possible subfactors by examining their discriminant validity in relation to trauma symptoms.

## Method

### Participants and Procedure

#### Clinical sample

The clinical sample consisted of 1,791 outpatients of Foundation Centrum ’45, a specialized Dutch center for treatment and diagnosis of complex psychotrauma. Data collection took place between January 2001 and May 2012. About 60% of the sample consisted of Dutch patients (mainly police officers, military veterans, and World War II survivors and their children). The remaining 40% of the sample consisted of refugees who had temporary or permanent refugee status or Dutch nationality and whose language proficiency was sufficient to complete a diagnostic assessment in Dutch. Refugees mainly came from the Middle East, Sub‐Saharan Africa, and Balkan Europe. Most patients at Foundation Centrum ’45 had been diagnosed with PTSD and/or depressive disorder. The WAS was self‐administered as part of a routine diagnostic assessment in all patients who applied for treatment at Foundation Centrum ’45. The responses analyzed in this paper were collected before the start of treatment. Respondents were between 21 and 88 years of age, with a mean age of 52.15 years (*SD =* 12.84). The majority were men (59.3%). A local institutional review board was consulted to review the ethical merits of the current study and stated that no review of the ethical merits of the study was needed because only secondary data analysis was conducted on questionnaires that were administered for diagnostic purposes within the institution. Patients gave informed consent to use their data in scientific research.

#### Healthcare professionals

The second sample consisted of 236 healthcare professionals, mainly psychotherapists, nurses, and doctors, who filled out the questionnaire as part of an educational program on trauma treatment. A total of 36 respondents had missing values for age, and 47 answered a variant of the age question that used broad categories. The other 153 respondents were between 23 and 76 years old with mean age of 48.95 years (*SD* = 9.44). A majority of participants in this sample were women (56.4%), and 13 respondents had missing data in terms of gender.

### Measures

#### Assumptions about the world

The WAS is a self‐report questionnaire consisting of 32 items regarding assumptions about the world (Janoff‐Bulman, 1989). The items theoretically form eight subscales that consist of four items each. Answers are given on a 6‐point scale ranging from 1 (*strongly disagree)* to 6 (*strongly agree)*. The Dutch version we used was the result of a forward‐backward translation procedure as well as consultation with a language professional. Items 3, 6, 8, 15, 18, 24, and 31 are reverse‐scored (from this point on indicated with an “R” next to the item number). Contrary to the original version, Items 2 and 12 were positively formulated, and items were presented in clusters according to the eight subscales. This last adaptation was made to facilitate use of the paper‐and‐pencil version in clinical practice (Kleijn & Smith, 1999). To avoid confusion, we used the item numbers from the original version of the questionnaire in this paper. Cronbach's alpha values for the subscales ranged from .73 to .84 (see Table 2).

**Table 2:** Pattern Matrix of Component Loadings Resulting From Factor Analysis With GEOMIN Rotation

	Benevolence of the Impersonal World^a^	Benevolence of the People	Justice	Self‐Worth	Randomness	Self‐Controllability	Luck	Controllability
1: Item 5	.73*							
2: Item 9	.84*							
3: Item 30	.55*							
4: Item 25	.39*	.38*						
5: Item 12		.47*						
6: Item 2		.84*						
7: Item 26		.87*						
8: Item 4		.64*						
9: Item 14			.67*					
10: Item 19		.36*	.72*					
11: Item 7			.45*					.36*
12: Item 1			.55*					
13: Item 8				.80*				
14: Item 18				.94*				
15: Item 31				.67*				
16: Item 28				.44*				
17: Item 3					(.20*)			
18: Item 6					.82*			
19: Item 15					.83*			
20: Item 24					.67*			
21: Item 13						.68*		
22: Item 17						.82*		
23: Item 23						.74*		
24: Item 27						.70*		
25: Item 10							.83*	
26: Item 16							.72*	
27: Item 21							.74*	
28: Item 32							.81*	
29: Item 11								.56*
30: Item 20								.74*
31: Item 22								.63*
32: Item 29								.67*
Eigenvalue	7.22	3.36	2.71	2.19	2.00	1.75	1.34	1.13
Cronbach's α	.80	.83	.76	.77	.68	.79	.84	.73

*Note. n* = 597; Subsample 1, outpatients of a trauma treatment center. Factor loadings below .35 were suppressed for reasons of clarity.

^*^ *p* < .05.

#### Traumatic experiences

We used the sections of the Harvard Trauma Questionnaire (Mollica et al., 1992) that deal with trauma events (HTQ Events) and trauma symptoms (HTQ Symptoms). The HTQ Symptoms and HTQ Events assessments were administered only to participants in the clinical sample who were refugees (*N* = 266). The HTQ Events portion consists of 20 items that give descriptions of traumatic events, such as “imprisonment,” “lack of food or water,” and “forced separation from family/relatives.” Each description has four answer categories: *no*, *heard about*, *witnessed*, and *experienced*. We rescored the HTQ Events section in a dichotomous way, distinguishing between *experienced* and all other answer categories. The HTQ Symptom section consists of 16 trauma symptoms derived from the revised third edition of the *Diagnostic and Statistical Manual for Mental Disorders* (*DSM III‐R*) criteria for PTSD, such as “recurrent nightmares,” “difficulty concentrating,” and “the feeling of having no future.” Two of the *DSM‐III‐R* symptoms, namely intense psychological distress at exposure to events (B4) and physiological activity upon exposure to events (D5), are collapsed into one question. For each symptom, respondents are asked to rate the extent to which this symptom has bothered them in the previous week on a 4‐point scale, with answer categories ranging from 1 (*not at all*) to 4 (*extremely*); and the total score is an average of the ratings for each symptom. The Cronbach's alpha value for the HTQ Symptom section was .94.

### Data Analysis

We performed a stepwise analysis of the factor structure of the WAS in three random subsamples, each consisting of 597 participants, of the total patient sample. We split the sample into three subsamples in order to perform an EFA in the first subsample, a CFA in the second subsample and then to test the final model with a CFA in the third subsample. Missing values on WAS items were present for 15.6% of the cases, ranging from missingness of 0.5% (Item 5) to 3.0% (Item 15) on the individual items. These values were imputed for each dataset separately using the expectation maximization algorithm in SPSS (Version 24.0). EFA and CFA were performed using Mplus 7.11 (Muthén & Muthén, 1998–2012). First, we conducted weighted least squares mean and variance (WLSMV) adjusted EFA on one‐third of the patient sample (Subsample 1); we chose WLSMV given the ordinal level of the data. Because we expected correlations between the different factors, an oblique (GEOMIN) rotation was used, and because parallel analysis (Horn, 1965) is not available for WLSMV in Mplus, we repeated the EFA using robust maximum likelihood estimation and applied the parallel test to further substantiate our choice for an EFA model. The selected model, based on scree plot (Cattell, 1966), criterion for eigenvalues greater than 1 (Kaiser, 1960), and parallel analysis (Horn, 1965), was tested with WLSMV CFA in a second part of the patient sample (Subsample 2). To examine the justification of scoring of subscales, their possible interrelatedness, and the possible influence of a single general underlying factor, we compared three models, namely the correlated model that followed from the EFA, a strict unidimensional model, and a bifactor model in which both the influence of a general factor and the specific factors (all uncorrelated) of our EFA are shown. Bifactor modeling is helpful in getting more insight in the relative influence of a general factor and the role of specific factors after partialling out this general factor. This may, for example, be important in the decision to use total scores and/or form subscale scores. Strong loadings on the general factor that are not much lower than those in the unidimensional model and small and/or nonsignificant loadings on the specific factors would suggest unidimensional scoring (Chen, Hayes, Carver, Laurenceau, & Zhang, 2012; Reise, Morizot, & Hays, 2007). Additionally, we calculated the explained common variance (ECV), defined as the ratio of variance explained by the general factor divided by the variance explained by the general plus the specific factors (Rodriguez, Reise, & Haviland, 2006). Higher ECV values indicate a strong general factor, with values greater than .60 suggested as a tentative benchmark for sufficient unidimensionality (Reise, Scheines, Widaman & Haviland, 2013).

Different fit indices were used in CFA to evaluate these three factor models, including chi‐squares (with lower values indicative of better fit) and the ratios of the chi‐square to its degrees of freedom (*df;* i.e., χ^2^/*df*). There are no absolute standards for the ratio of chi‐square to degree of freedom, but ratios close to or less than 2 are considered to represent good fit and ratios less than 5 (Watkins, 1989) or 3 (Schermelleh‐Engel, Moosbrugger, & Müller, 2003) are considered to represent acceptable fit. Several additional indices were selected, following the recommendations of Hu and Bentler (1998); the comparative fit index (CFI), the Tucker‐Lewis Index (TLI), and the root mean square error of approximation (RMSEA). For TLI and CFI, values greater than or equal to .90 and .95 are considered indicative of acceptable and good model fit, respectively. For the RMSEA values less than .08 and .06, respectively, are considered to reflect acceptable and good model fit (Browne & Cudeck, 1993; Hu & Bentler, 1999). Differences in fit between nested models were statistically tested using the Mplus DIFFTEST procedure, which appropriately computes differences in chi‐square values of nested models. Next to this, we compared CFI and RMSEA values, using a cut‐off point of greater than .01 for change in CFI values (Cheung & Rensvold, 2002) and greater than .015 for change in RMSEA values (Chen, 2007). The best‐fitting model was cross‐validated in the third part of our clinical sample (Subsample 3).

The theory of shattered assumptions claims to apply to general populations as well as clinical groups; we therefore examined whether the model that was found in the clinical sample could be replicated in the sample of healthcare professionals. In this sample, missing values were found for 6.3% of the cases, with a maximum of 1.1% missingness (Items 27, 28, and 32) for individual items. These values were also imputed using the expectation maximization algorithm in SPSS. Automatic multigroup factor analysis, introduced in Mplus 7.1, was used to check measurement invariance between Subsample 3 and the healthcare professionals sample on three increasingly restrictive levels: configural invariance (same factor structure), metric invariance (same factor structure and equal factor loadings), and scalar invariance (same factor structure and equal factor loadings and thresholds). Because chi‐square difference tests are sensitive to sample size (Chen, 2007), this value was only used for descriptive purposes, and the absence of relevant changes in RMSEA (ΔRMSEA > .015) and CFI values (ΔCFI > .01) between increasingly restrictive models was seen as evidence for sufficient measurement invariance.

Following the results of our factor analysis, we did a univariate regression analysis on the relationship between the scores of the WAS subscales, the number of traumatic events as measured by HTQ Events section and the severity of trauma symptoms as measured by the HTQ Symptoms section, and a multivariate regression analysis for the associations among WAS subscales and the severity of trauma symptoms after controlling for the number of traumatic experiences. The aim of this analysis was to study the incremental validity of the different WAS factors. Missing values for HTQ Events items were found in 25.6% of the cases, with item‐specific missingness ranging from 1.2% (Items 10 and 17) to 7.5% (Item 20). Missing values for HTQ Symptoms items were found in 8.6% of the cases, with item‐specific missingness ranging from 0.8% (Items 3 and 8) to 2.6% (Items 4, 11, and 12). These values were again imputed using the expectation maximization algorithm in SPSS.

## Results

Results of the EFA performed with data from Subsample 1 *(n =* 597) of the clinical sample are given in Table 2. Eigenvalues and the scree plot clearly indicated an eight‐factor solution, which almost perfectly resembled the hypothesized structure (see Table 2). Only Item 3R was not clearly associated with one of the factors. When we applied an arbitrary threshold of greater than .35 for factor loadings, a cross loading was found for Items 7, 19, and 25. Three significant (*p* < .05) factor correlations with moderate (*r*s = .35–.48) strength were found, with the strongest correlation between items representing Benevolence of the World and Benevolence of the People, *r* = .48, *p* < .05. The parallel analysis supported a model of seven factors, with the eigenvalue of the eighth factor just below the corresponding eigenvalue in the random data. In this model of seven factors, the items in both Benevolence factors grouped together. However, we chose to test the eight‐factor solution with CFA because it resembled the underlying theory, and the results of the scree test and eigenvalue criterion were supportive of this model.

We tested the eight‐factor solution with CFA against a unidimensional and a bifactor model in Subsample 2 (i.e., the second part of the clinical sample). As can be seen in Table 3, fit indices did not provide support for the unidimensional model, but fit indices for the model with eight correlated factors and the bifactor model were within the acceptable range (with the exception of the TLI value for the bifactor model), which was just below the threshold for acceptable fit. Given the more restricted character of the TLI, weaker fit indices could be expected for the correlated eight‐factor model compared to the bifactor model, but this was not the case; this finding provides further support for the correlated eight‐factor model. This support was also illustrated by the fact that the majority of the items in the bifactor model (65.6%) loaded more strongly on their specific factor compared to the general factor (a graphical representation of the bifactor model can be requested from the first author). The ECV of the general factor was only 0.33, far below the benchmark of 0.60 for sufficient unidimensionality, and indicated that 67.0% of the common variance spread across the eight specific factors. Factor analysis thus was not supportive of an (essentially) unidimensional model, but supported the importance of a model of different factors. Because the eight‐factor model was in line with the underlying theory and the fit indices for this solution were acceptable, we cross‐validated this model in Subsample 3 (i.e., the third and final part of the clinical sample), which resulted in very similar acceptable fit indices. As is shown in Table 4, the final model had three moderate (greater than .3) and five strong (greater than .5) factor correlations, which shows that there are large differences in the associations between factors: Some are strongly related whereas no significant associations between others could be substantiated. The original clustering of factors in three domains does not account for these differences (Table 1) as only three of the eight moderate‐to‐strong correlations were found between factors of one domain. Subscales had acceptable internal consistencies, Cronbach's alpha values ranging from .68 till .84 (Table 2). Finally, we tested the eight‐factor model in our sample of healthcare professionals, which resulted in acceptable fit indices (see Table 3). As shown by the results of our analysis of measurement invariance (Table 5), both CFI and RMSEA values did not show a relevant decrease in fit between increasingly restrictive models, which supports full scalar measurement invariance of the WAS between the samples of patients' healthcare professionals. This means that for both populations, items were associated in the same way to the eight‐factor model and that also the levels of the underlying items were equal in both groups, which made the comparison of mean scores possible. To examine the influence of data imputation, we repeated EFA and CFA with nonimputed data, and this resulted in strongly comparable results.

**Table 3:** Model Fit Indices for Three Models Tested in Confirmatory Factor Analysis

Model	χ^2^	*df*	χ^2^/*df*	RMSEA	90% CI	CFI	TLI
1‐factor	9,841.13***	464	21.21	0.18	[0.18, 0.19]	0.45	0.41
Correlated 8‐factor	1,832.06***	436	4.20	0.07	[0.07, 0.08]	0.92	0.91^a^
Bifactor	2,092.64***	432	4.84	0.08	[0.08, 0.08]	0.90	0.89
Correlated 8‐factor^b^	1,710.06***	436	3.92	0.07	[0.07, 0.07]	0.92	0.91
Correlated 8‐factor^c^	807.06***	436	1.85	0.06	[0.05, 0.07]	0.92	0.91

*Notes. n* = 597 for both subsample 2 and subsample 3, outpatients of a trauma treatment center, and *n* = 236 for healthcare professionals. *df* = degrees of freedom; RMSEA = root mean square error of approximation; CFI = comparative fit index; TLI = Tucker–Lewis Index.

^a^Significantly better fit than one‐factor model: χ^2^(28) = 3,218.35, *p* < .001.

^b^Cross validation Subsample 3.

^c^Cross validation healthcare professionals sample.

^***^ *p* < .001.

**Table 4:** Matrix of Factor Correlations for Correlated Eight‐Factor Model

	Benevolence of the People	Justice	Self‐Worth	Randomness	Self‐Controllability	Luck	Controllability
Benevolence of the Impersonal World	.67***	.53***	.30***	.15***	.10*	.55***	.04
Benevolence of the People	−	.59***	.27***	.18***	.11*	.51***	.16***
Justice		−	.12**	.16***	.13**	.47***	.50***
Self‐Worth			−	.28***	.17***	.41***	−.02
Randomness				−	−.22***	.09*	−.04
Self‐Controllability					−	.28***	.30***
Luck						−	.17***

*Note*. *n* = 597 for Subsample 2, outpatients of a trauma treatment center.

^*^ *p* < .05. ^**^ *p* < .01 ^***^ *p* < .001.

**Table 5:** Measurement Invariance (MI) Between Patient Sample and Healthcare Professionals Sample

MI Level	χ^2^	*df*	RMSEA	90% CI	CFI	TLI	Δχ^2^	*df*
Configural	2,377.81	872	0.06	(0.061, 0.067]	0.93	0.92		
Metric	2,415.08	896	0.06	[0.061, 0.067]	0.92	0.92	102.03	24^a^
Scalar	2,634.73	1016	0.06	[0.059, 0.065]	0.92	0.92	390.45	120^a^

*Note. n* = 597 for Subsample 3, outpatients of a trauma treatment center, and *n* = 236 for healthcare professionals. *df* = degrees of freedom; RMSEA = root mean square error of approximation; CFI = comparative fit index;

a *p* < .0001 compared to the configural model.

To study the incremental validity of the eight‐factor model of the WAS, we carried out a regression analysis with trauma symptoms as the dependent variable on a subsample of refugees who completed the HTQ (*n* = 266). For the HTQ Symptoms section, a mean score of 3.06 (*SD =* 0.57) was found, and 77.8% of those who took the assessment had a mean score above 2.5, which is seen as the cut‐off indicating likelihood of PTSD (Mollica et al., 1992). We also rescored the HTQ Symptoms items in a dichotomous way, using the rating choices *quite a bit* and *all the time* to represent endorsement; after this rescoring, a total of 80.8% of our respondents now met *DSM III‐R* Criteria B, C, and D for PTSD. For the HTQ Events subsection, a median score of 17 was found when all response categories other than *no* were taken together, which meant that 50% of participants responded affirmatively (i.e., selected *heard about* or *witnessed or experienced* rating options) to 17–20 various traumatic events. For the response category *experienced*, the median score was 11. The traumatic events that were most frequently affirmed as having been experienced were “almost died” (74.4%), “other” (68.0%), and “being threatened with physical torture” (66.2%). The least frequently affirmed events were “rape or sexual abuse” (17.3%) and “murder of stranger” (39.1%). As can be seen in Table 6, univariate regression analysis showed significant associations with the WAS factors Self‐Worth, Benevolence of the World, Benevolence of People, Justice, and Luck. Multivariate regression analysis on the WAS subscales showed that the number of traumatic experiences as measured by the HTQ Events subsection explained 12.2% of the variance in trauma symptoms as measured by the HTQ Symptoms subsection. After entry of the WAS scales, the total variance explained was 31.0%, *F*(9, 256) = 12.79, *p* < .001. The WAS subscales explained an additional 19.0% of the variance in trauma symptoms after controlling for the number of traumatic experiences, *∆R* ^2^ = .19, ∆*F*(8, 256) = 8.73, *p* < .001. In the multivariate regression analysis, 2 of the 8 WAS factors showed significant beta values: Self‐Worth, β = −.31, *p* < .001; and Luck, β = −.15, *p =* .020. This means that they explained additional variance in trauma symptoms in addition to the variance that was explained by the number of traumatic events and the part they shared with the other WAS factors.

**Table 6:** Regression of Trauma Severity on Traumatic Events and World Assumption Scale (WAS) Factors

	Univariate		Multivariate	
Variable	β	*t*(264)	β	*t*(256)
HTQ Events^a^	.35	6.05***	.25	4.61***
Benevolence of the Impersonal World	−.28	−4.68***	−.10	−1.51
Benevolence of the People	−.28	−4.69***	−.06	−0.89
Justice	−.17	−2.79*	−.09	−1.38
Self‐Worth	−.38	−6.65***	−.31	−5.40***
Randomness	−.08	−1.27	−.00	−0.04
Self‐Controllability	−.01	−0.11	.10	1.83
Luck	−.32	−5.40***	−.15	−2.46*
Controllability	−.03	−0.52	.03	0.55

*Note. n* = 266, outpatients of a trauma treatment center. HTQ = Harvard Trauma Questionnaire.

a Model 1 (HTQ Symptoms): Adjusted *R* ^2^ = .12; *F*(1, 264) = 36.63, *p* < .001. Model 2 (HTQ Symptoms and WAS factors): Adjusted *R* ^2^ = .29; ∆*R* ^2^ = .19; *F*(8, 256) = 8.73, *p* < .001.

^*^ *p* < .05. ^***^ *p* < .001.

## Discussion

Janoff‐Bulman (1992) developed the WAS as a measure of eight positive assumptions about the benevolence of people and the world, the meaningfulness of events, and self‐worth. According to the underlying theory of shattered assumptions, a change in these assumptions is an important aspect of posttraumatic stress. The WAS has been examined in many studies, with mixed results regarding its validity, and there is an ongoing discussion regarding its factor structure. The use of different factor solutions limits the interpretation of results and comparison between studies. The results of the current study consistently show acceptable fit for the original eight‐factor structure in both the clinical sample and the sample of healthcare professionals. Fit indices were better for this model in comparison to those for a unidimensional and even a bifactor model, and all factors showed acceptable or near‐acceptable internal consistencies.

These results are in line with the original EFA (Janoff‐Bulman, 1992) and with a CFA that was performed on a clinical sample (Elklit et al., 2007), but they are not consistent with the results of a CFA performed by Kaler et al. (2008) on a student sample nor with several other EFAs. A difference between our study and previously conducted EFAs is that we performed this analysis on a clinical sample whereas most former studies used student samples. Rini et al. (2004) did use a clinical sample, but they only included 100 respondents, which may be seen as too small for an EFA on a measure with 32 items. The differentiation between world assumptions may be more pronounced in a clinical sample than in a student sample. As far as we know, our study was the first to analyze the WAS and also study measurement invariance. Because the theory behind the WAS is meant to apply to both general and clinical populations, it is important that the WAS can be used in samples that represent both. Our findings showed that the factor structure, loadings of the items onto these factors, and thresholds were the same for both the clinical and healthcare professional samples; this made it possible to use the WAS to make comparisons between the samples.

As an initial evaluation of the differential role of positive assumptions as measured by the WAS in posttraumatic symptomatology, we performed a regression analysis. Of all the WAS factors, Self‐Worth turned out to have the strongest unique association with posttraumatic complaints. This is in line with previous research on the WAS and the wide recognition of self‐worth as an important factor in the development of psychopathology (Zeigler‐Hill, 2011). We also found a unique, although small, association between Luck and posttraumatic symptoms, for which we found no clear explanation in previous literature. The effect may have been specific for the respondents in the subsample we used for this analysis, all of whom were refugees. Additionally, it is important to note that Luck belongs to the same domain as Self‐Worth, and that its items are also about the self‐image of the person. The differences we found between different factors and their associations with trauma symptoms illustrate the usefulness of distinguishing between assumptions and their association with trauma measures.

Data collection for the clinical sample took place during a period of 11 years. This was disadvantageous in that there were changes in the battery of administered questionnaires during this long period and it was not possible to fill in incomplete demographic data. However, a strength of the study was that data were collected in a sample of people who had requested help for posttraumatic complaints, which favors the external validity of our clinical data. Our sample of healthcare professional was a convenience sample as these respondents participated as part of a course in treatment methods. Therefore, the sample of healthcare professionals cannot be seen as representative of the general population. Moreover, we cannot exclude the possible presence of symptomology in this sample. Previous studies (e.g., Dattilio, 2015) have suggested that healthcare professionals might experience more mental health complaints compared to the general population, and this might have resulted in a WAS factor structure that is more comparable to the results of our clinical sample. Confirmation of our findings in additional samples is recommended.

Although this study supported the model that contained eight related factors, it should be noted that fit indices were only acceptable and that strong interrelations were found for several factors. These strong interrelations are not unexpected given the conceptual overlap between assumptions, such as between both benevolence factors, and between Controllability and Self‐Control (see Table 1). It should also be noted that we used the WAS in a clustered format. Therefore, comparisons with former research are only possible to some extent. Our clustered format may be an important reason for we found evidence supporting the model of eight related factors whereas former factor analytic studies showed mixed results. It has long been recognized that the context of items, including the order in which they are presented, may greatly influence responses (Tourangeau & Rasinski, 1988). A disadvantage of a clustered format, as was used in this study, might be that respondents consider the content of the different related items less seriously when they are placed together compared to when they are presented at random. On the other hand, clustering decreases the risk of giving unintended responses. However, the effect of item clustering is debated, and experimental studies with other questionnaires have yielded conflicting results. When choosing a format, it is important to note whether scores of subscales or total scores only will be relevant on conceptual grounds. Because the differentiation between assumptions is important in the conceptualization underlying the WAS, it may be advisable to use a clustered format in future studies.

Although our study supported the usefulness of the WAS in examinations of the theory of shattered assumptions, it is important to note that its factorial validity can still be improved. For example, some of the WAS items turned out to be only weakly associated with a factor or to be associated with different factors, as shown in Table 2. Both Benevolence factors turned out to be strongly associated with one another and perhaps should not be considered as distinct factors, which is also in line with the EFA findings reported by Janoff‐Bulman (1989).

Research on the theory of shattered assumptions has primarily focused on changes in different assumptions in relation to experienced traumatic events and has greatly neglected the possible interactions between different assumptions and between different assumptions and other constructs. It may, for example, be hypothesized that the experience of social support in the aftermath of a traumatic event may lead to increased trust in the Benevolence of People while at the same time, the trust in Meaningfulness of Events or Self‐Worth decreases. More focus on the role of different assumptions, and especially in longitudinal studies and in terms of associations with different types of traumatization, may help to better understand the process of posttraumatic adaptation and the development of mental health disorders. Our study showed that the WAS differentiates between the assumptions of the theory of shattered assumptions and thus might be useful in future studies regarding the role of cognitive changes in the aftermath of traumatic events. It might also have relevance for clinical practice by helping guide therapists to focus on assumptions that need to be addressed in therapy. A change in basic assumptions has long been recognized as one of the possible effects of experiencing traumatic events, and focusing on this aspect seems important for understanding and helping people with posttraumatic complaints.
